# Detection of Hepatic Metastasis in Colorectal Cancer: A Comparative Case Report of ^18^F-FDG PET-CT and Diffusion-Weighted MRI with a b Value of 1000

**DOI:** 10.5334/jbsr.3186

**Published:** 2023-07-10

**Authors:** Andrei-Cristian Fülöp, Gabriel Serac, Simona Gurzu

**Affiliations:** 1 Department of Radiology and Imaging, George Emil Palade University of Medicine, Pharmacy, Science and Technology, Targu Mures, Romania; 2 Department of Surgery, George Emil Palade University of Medicine, Pharmacy, Science and Technology, Targu Mures, Romania; 3 Department of Pathology, George Emil Palade University of Medicine, Pharmacy, Sciences and Technology, Targu Mures, Romania

**Keywords:** colorectal cancer, liver metastasis, PET-CT, MRI, diffusion-weighted imaging

## Abstract

Detection of hepatic metastasis from colorectal cancer remains a clinical challenge. In this case report, we present a 66-year-old male patient with a rectal carcinoma who underwent ^18^F-FDG PET-CT for staging, which revealed one hepatic metastasis. Abdominal magnetic resonance imaging (MRI) with diffusion-weighted sequence with a b value of 1000 was performed, which identified a second metastasis of the liver.

**Teaching Point:**

This case report illustrates that, in some patients, diffusion-weighted MRI with a b value of 1000 might be a more sensitive technique for detecting small hepatic metastases than ^18^F-FDG PET-CT.

## Introduction

Colorectal cancer (CRC) most frequently develops in the sigmoid colon, accounting for 35–50% of all cases, followed by the upper rectum, which represents approximately 20% of cases [1]. Imaging plays an essential role in the staging and monitoring of this disease to guide and evaluate the efficacy of treatment. CRC has a high tendency to metastasise to the liver, and detecting hepatic metastases is critical for prognosis and treatment. Flourine-18 fluorodeoxyglucose positron emission tomography/computed tomography (^18^F-FDG PET-CT) is frequently used in cancer imaging and staging; however, it has limited sensitivity for detecting small hepatic metastases. In contrast, magnetic resonance imaging (MRI) can detect smaller hepatic metastases but has lower specificity [2,3]. The recent introduction of high b value diffusion sequences to MRI has significantly improved the detection of hepatic metastases by evaluating water motion at the cellular level. In this case report, we present a patient with upper rectal cancer in whom ^18^F-FDG PET-CT failed to identify a hepatic metastasis, while abdominal MRI with diffusion-weighted sequence (DWI) with a b value of 1000 identified all metastases, which were subsequently confirmed intraoperatively and histopathologically. This paper highlights the usefulness of high b value DWI MRI for detecting small hepatic metastases and their relevance for guiding treatment and monitoring the disease.

## Case History

**Figure 1:**
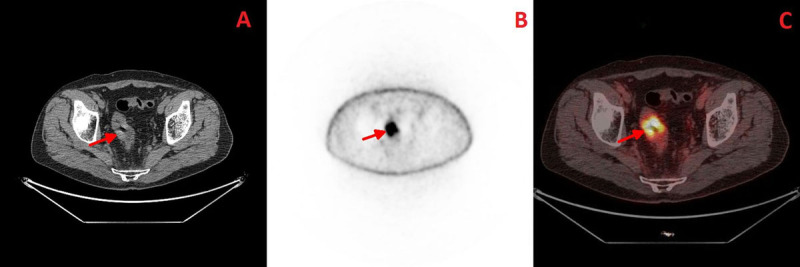
Axial plane; **A-** CT image highlighting the rectal tumour; **B-** PET image; **C-** Fused PET-CT image.

**Figure 2:**
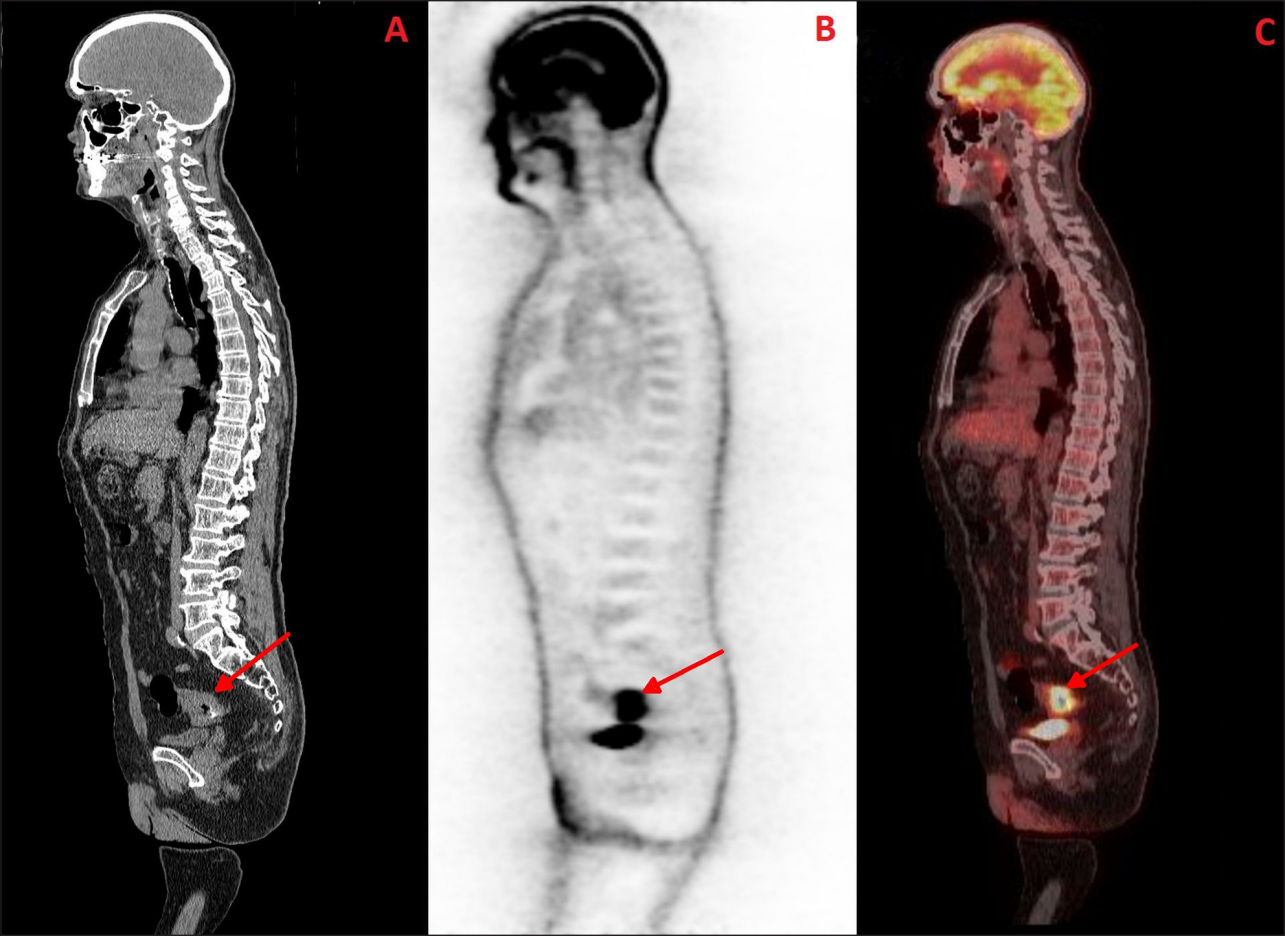
Sagittal plane; **A-** CT image; **B-** PET image; **C-** Fused PET-CT image.

**Figure 3:**
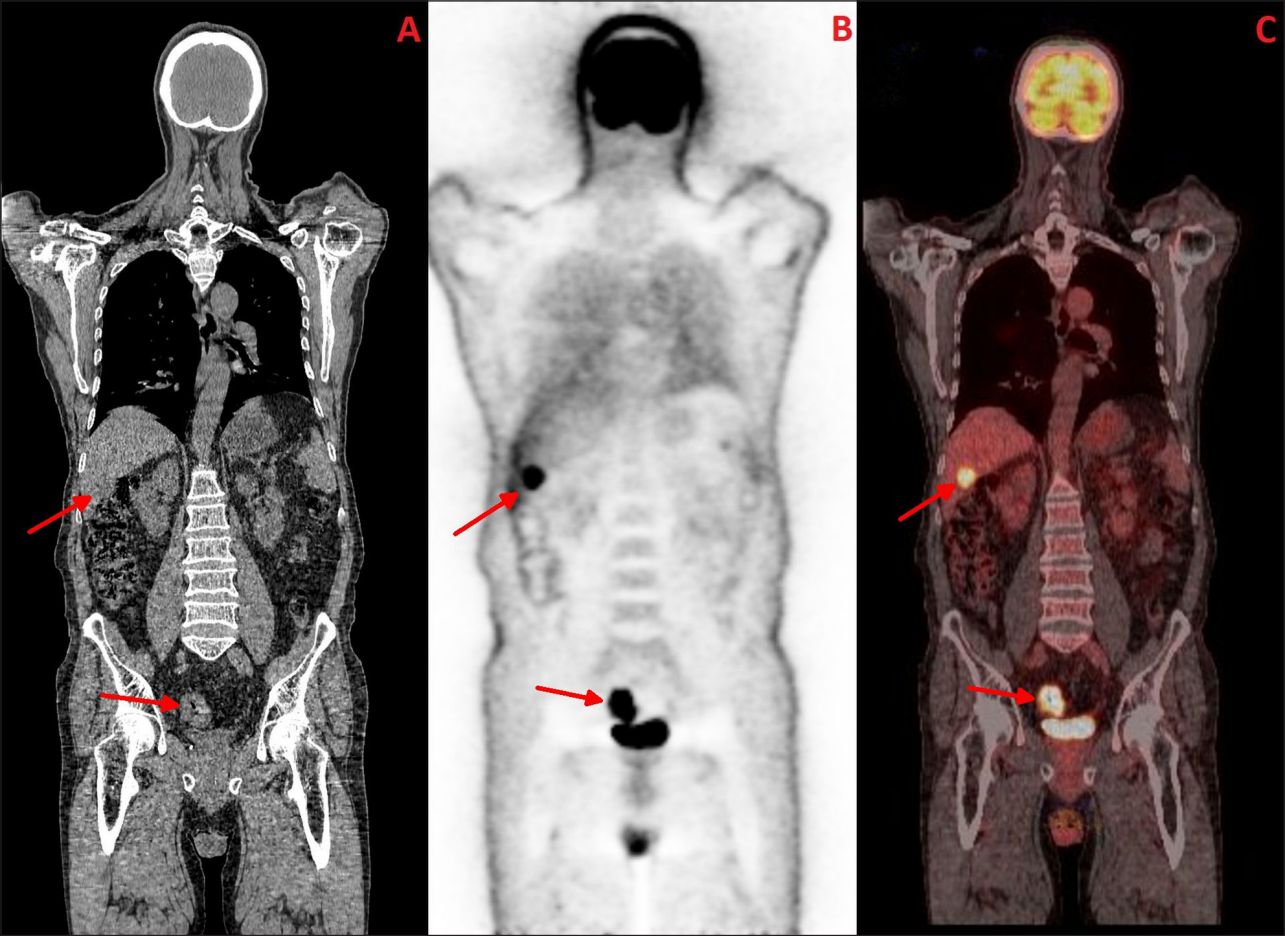
Coronal plane; **A-** CT image revealing the rectal and liver lesions; **B-** PET image; **C-** Fused PET-CT image.

A 66-year-old male patient was admitted to hospital with non-specific gastrointestinal symptoms, namely chronic constipation. He had no significant medical history, and physical examination was unremarkable. As the patient presented with anaemia, which was presumed to be caused by an occult haemorrhage, colonoscopy was indicated. It emphasized a tumour mass in the upper rectum which, after biopsy, was concluded to be a microsatellite stable BRAF-wild-type adenocarcinoma displaying KRAS-mutations (12ASP). ^18^F-FDG PET-CT staging revealed focal uptake in the upper rectum (Figures 1 and 2), with only one distant, 35-mm metastatic lesion in the anterior lateral segment of the liver (Figure 3).

**Figure 4:**
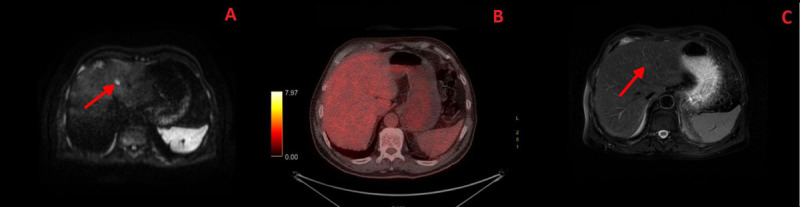
Axial plane; **A-** Diffusion-weighted MRI image with a b value of 1000; **B-** Fused PET-CT image; **C-** T2-weighted MRI.

Given the increased risk of other distant metastases in colorectal cancer, a surveillance MRI using a 1.5 Tesla GE Optima 450W system was performed. Abdominal-pelvic MRI with DWI with a b value of 1000 revealed the lesions identified by ^18^F-FDG PET-CT; however, it further identified numerous affected lymphatic nodules and another suspicious 12-mm lesion with restricted diffusion in the left medial segment of the liver (Figure 4A). This lesion was consistent with another hepatic metastasis that had not been identified by ^18^F-FDG PET-CT (Figure 4B). The lesion was barely visible on the T2-weighted sequences due to its isointense signal (Figure 4C).

After three months of neoadjuvant chemoradiotherapy, colectomy with resection of the upper rectum and liver metastasectomy was done. Postoperative histopathological examination of the resected specimen revealed a moderately differentiated adenocarcinoma and metastases in six regional lymph nodes, as seen on MRI. Both metastatic lesions in the liver were confirmed histopathologically. The patient was subsequently referred for adjuvant chemotherapy and remained disease-free on clinical and radiological follow-up for three months after surgery.

## Comments

This case report provides strong evidence supporting the higher sensitivity of DWI MRI compared to ^18^F-FDG PET-CT in detecting small hepatic metastases from CRC, although further studies are needed to draw definitive conclusions [4,5]. Some tumours may not exhibit significant increase in glucose metabolism, or the increase may be too subtle to detect. In addition, PET-CT may not be able to detect small tumours, especially those that are less than 1 cm in diameter, due to the limitations of its image resolution. As a result, DWI MRI has emerged as the most precise imaging technique for evaluating colorectal liver metastases.

In patients with CRC, multimodal imaging techniques might be useful for detecting and staging of cases with high risk of liver and peritoneal metastases. Our case demonstrates the clinical relevance of this recommendation and the benefits of early detection of hepatic metastases.

## Conclusion

In patients with rectal cancer, MRI with high b value DWI can successfully detect small metastatic lesions of the liver that may not be detected by ^18^F-FDG PET-CT. This cost-effective MRI-based technique can be used as a surrogate method for pre-operative assessment and post-operative follow-up.
